# Codon Optimization Enables the Geneticin Resistance Gene to Be Applied Efficiently to the Genetic Manipulation of the Plant Pathogenic Fungus *Botrytis cinerea*

**DOI:** 10.3390/plants13020324

**Published:** 2024-01-22

**Authors:** Maoyao Tang, Yangyizhou Wang, Kexin Wang, Yuanhang Zhou, Enshuang Zhao, Hao Zhang, Mingzhe Zhang, Hang Yu, Xi Zhao, Guihua Li

**Affiliations:** 1 College of Plant Sciences, Jilin University, Changchun 130062, China; tangmy21@mails.jlu.edu.cn (M.T.); wyyz17602868149@163.com (Y.W.); kxwang22@mails.jlu.edu.cn (K.W.); mzzhang@jlu.edu.cn (M.Z.); yuhang8220@mails.jlu.edu.cn (H.Y.); zhaoxi8220@mails.jlu.edu.cn (X.Z.); 2 Research Management Department, Changchun Academy of Forestry, Changchun 130021, China; meyuanhang@163.com; 3 College of Computer Science and Technology, Jilin University, Changchun 130012, China; zhaoes22@mails.jlu.edu.cn (E.Z.); zhangh@jlu.edu.cn (H.Z.)

**Keywords:** grey mould disease, genetic transformation, positive selection, *NPTII*

## Abstract

*Botrytis cinerea* can infect almost all of the important horticultural crops and cause severe economic losses globally every year. Modifying candidate genes and studying the phenotypic changes are among the most effective ways to unravel the pathogenic mechanism of this crop killer. However, few effective positive selection markers are used for *B. cinerea* genetic transformation, which limits multiple modifications to the genome, especially genes involving redundant functions. Here, we optimized a geneticin resistance gene, *BcNPTII*, based on the codon usage preference of *B. cinerea*. We found that *BcNPTII* can greatly increase the transformation efficiency of *B. cinerea* under G418 selection, with approximately 30 times higher efficiency than that of *NPTII*, which is applied efficiently to transform *Magnaporthe oryzae*. Using the gene replacement method, we successfully knocked out the second gene *BOT2*, with *BcNPTII* as the selection marker, from the mutant Δ*oahA*, in which *OAHA* was first replaced by the hygromycin resistance gene *HPH* in a field strain. We obtained the double knockout mutant Δ*oahA* Δ*bot2*. Our data show that the codon-optimized *BcNPTII* is an efficient positive selection marker for *B. cinerea* transformation and can be used for various genetic manipulations in *B. cinerea*, including field wild-type strains.

## 1. Introduction

*Botrytis cinerea* can infect over 1400 plant species, causing grey mould disease [1]. It mainly affects dicotyledonous plants and is a major concern in horticultural crop protection [2]. Grey mould is one of the most serious plant diseases in greenhouse production. The economic losses caused by this disease amount to between USD 10 and 100 billion worldwide annually [3,4]. Additionally, *B. cinerea* is listed as one of the top 10 plant pathogenic fungi based on its scientific and economic importance [5]. Therefore, *B. cinerea* has become an important model pathogenic fungus for studying the pathogenicity of highly aggressive necrotrophic fungi and their interaction mechanisms with hosts [5]. *B. cinerea* mostly uses conidia as the source of primary infection and re-infection of the hosts and is spread through wind, rain, irrigation water, or agricultural operations [6]. Upon reaching the surface of host plants, the conidia germinate and form germ tubes under conditions of low temperature and high humidity. The tips of the germ tubes are induced by signals derived from the host surface and swell slightly and develop into appressoria, or they may undergo further branching and develop into more complex and powerful infection cushions to invade hosts [7]. *B. cinerea* synthesizes a variety of pathogenic factors to participate in disease, including cell wall-degrading enzymes, cutinase, toxins, enzymes against host immune reactions, sRNA, and metal-chelating proteins [3,8,9]. On the one hand, these factors cooperate to suppress the host’s defense response, and on the other hand, they also assist in the rapid killing of host cells and the decomposition of host tissues as nutrients. Genome sequencing has revealed that *B. cinerea* is predicted to contain more than 15,000 protein-coding genes, many of which have unknown functions, especially in host infection [10], and a few of them are expected to be set as alternative molecular targets for grey mould control after systematic functional analysis.

One of the most commonly used strategies for gene function analysis is genetic methods, including forward genetics and reverse genetics. To conduct genetic research, it is always necessary to introduce exogenous DNA fragments into *B. cinerea*, which requires a positive selection marker for screening transformants easily. Currently, the most commonly used selection marker is the hygromycin resistance gene *HPH* [3,11,12,13]. In addition, other positive selection markers reported for *B. cinerea* transformation include the bialaphos (or glufosinate) resistance gene *BAR* [3], the sulfonylurea resistance allele of the *Magnaporthe oryzae ILV1* gene [11], the fenhexamid resistance gene (Fen^R^) [14], and the nourseothricin resistance gene (Nat^R^) [14]. In fungi such as *M. oryzae*, the geneticin resistance gene *NPTII* has been widely used [15,16,17]. However, geneticin positive selection has not yet been established efficiently for the genetic transformation of *B. cinerea*.

In this study, we first attempted to use *NPTII*, the gene applied efficiently to *M. oryzae* transformation [17], as a positive selection marker for transforming *B. cinerea*. However, we found that the transformation efficiency was very low. Subsequently, we optimized the *NPTII* codon according to the usage preference of *B. cinerea*, and named it *BcNPTII*. We found that this synthetic gene significantly improved the transformation efficiency of *B. cinerea*. *BcNPTII* can also be used for gene double knockout (in combination with *HPH*). Notably, the new geneticin resistance gene can be efficiently applied to genetic manipulations of field-isolated wild-type *B. cinerea* strains. This study provides an efficient positive selection marker, *BcNPTII*, for the genetic manipulation of *B. cinerea*.

## 2. Results

### 2.1. Sensitivity Test of B. cinerea to Geneticin

**Figure 1:**
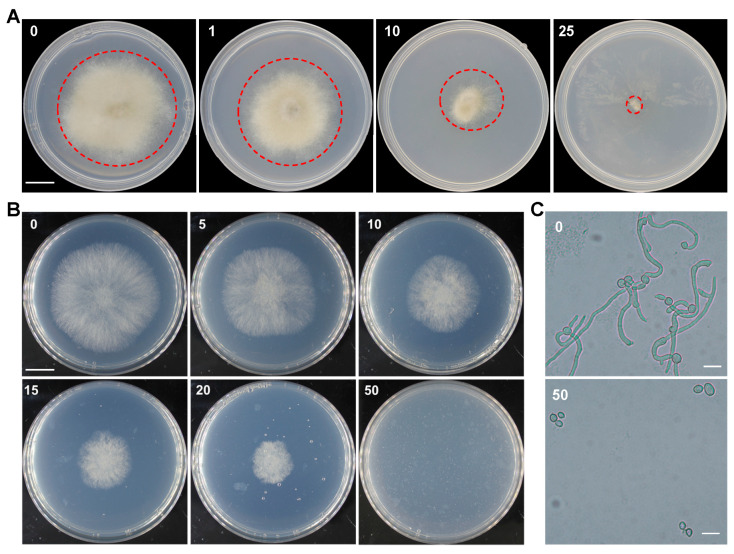
Sensitivity test of *Botrytis cinerea* to geneticin. (**A**) Mycelial plugs of B05.10 were inoculated on PDA plates supplemented with G418 at concentrations of 0, 1, 10, and 25 mg/L. Photographs were taken at 3.5 days post inoculation (DPI). 90 mm plates were used. Bar = 15 mm. Edges of the colonies are marked with red circles; (**B**) Conidial suspensions of B05.10 were inoculated on PDA plates supplemented with G418 at concentrations of 0, 5, 10, 15, 20, and 50 mg/L. Photographs were taken at 3 DPI. 70 mm plates were used. Bar = 15 mm. (**C**) Conidial germination in PDB media supplemented with or without G418 at 12 h post inoculation (HPI). Bar = 20 μm.

To establish an efficient geneticin genetic transformation system in *B. cinerea*, it is necessary to determine the appropriate concentration of antibiotic geneticin (G418) for selecting transformants. We performed a geneticin concentration gradient experiment to assess the sensitivity of *B. cinerea* to geneticin. Growth analysis of colonies formed on PDA plates supplemented with different concentrations of G418 showed that *B. cinerea* is very sensitive to G418 compared to the rice blast fungus. Using inoculation with mycelial plugs, we found that 10 mg/L G418 significantly inhibited the growth of the *B. cinerea* strain B05.10, and when the concentration was increased to 25 mg/L, the growth of B05.10 was completely suppressed, and the colonies were unable to expand from the inoculated agar plugs (Figure 1A). *M. oryzae* requires about 400 mg/L G418 for growth suppression.

Subsequently, we used the conidial inoculation method and tested the inhibitory effects of different concentrations of geneticin on conidial germination. Similar results to the mycelial plug inoculation experiment were obtained. We found that 10–20 mg/L G418 significantly inhibited the colony growth of B05.10, and no growth was observed when the G418 concentration was increased to 50 mg/L (Figure 1B and Figure S1). Microscopic observation revealed that 50 mg/L G418 completely inhibited the germination of *B. cinerea* conidia (Figure 1C). Based on the above analysis, we determined that 50 mg/L or higher G418 is the appropriate selection concentration for the genetic transformation of *B. cinerea*, and this concentration was used subsequently.

### 2.2. Codon Optimization of the Geneticin Resistance Gene NPTII Based on the Codon Usage Preference of B. cinerea

We first attempted to use *NPTII*, the gene applied efficiently to *M. oryzae*, to transform *B. cinerea*. However, the transformation efficiency was very low. We speculate that the codon usage of *NPTII* might not be consistent with that of *B. cinerea*. Codon analysis (http://www.kazusa.or.jp/codon/ (accessed on 8 August 2021)) showed that the coding GC content of the *B. cinerea* genome is 46.6%, which is significantly lower compared to that of the rice blast fungus at 56.3%. The two strains exhibit substantial differences in codon usage preference, which explains the efficient performance of *NPTII* in the rice blast fungus but lower effectiveness in *B. cinerea*. For example, the Arg codon CGC is used at a rate of 30.9% in the rice blast fungus and *NPTII*, but only 14.6% in *B. cinerea*. Similarly, the CGG codon is used at a rate of 13.9% in the rice blast fungus, but only 6.8% in *B. cinerea*. The Leu codon CTG is utilized at a rate of 28.9% in the rice blast fungus and *NPTII*, but only 10.7% in *B. cinerea*. Additionally, the Pro codon CCG is used at a rate of 26.1% in the rice blast fungus and *NPTII*, but only 11.4% in *B. cinerea*. The Thr codon ACG is used at a rate of 25% in the rice blast fungus and *NPTII*, but only 12.4% in *B. cinerea* (Table S1).

Since the codon usage of *NPTII* did not follow that of *B. cinerea* and was not suitable for the efficient transformation of this fungus, we subsequently optimized the codons of *NPTII* according to the usage preference of *B. cinerea*. A systematic codon optimization of the geneticin resistance gene *NPTII* was performed, following the strategy used for the codon optimization of the GFP gene in *B. cinerea* [18]. For each amino acid in the optimized gene, the top two most frequently used codons in *B. cinerea* were selected and differentially assigned based on their frequency of usage. Additionally, in the final CDS sequence, restriction endonuclease cleavage sites used frequently were removed (Table S1) to facilitate subsequent cloning. The new geneticin resistance gene optimized here was named *BcNPTII*, whose sequence was deposited in the Genbank database, and the accession number is OR921207.

### 2.3. The Codon-Optimized Gene BcNPTII Significantly Enhances the Transformation Efficiency of B. cinerea under the Selection Pressure of Geneticin

**Figure 2:**
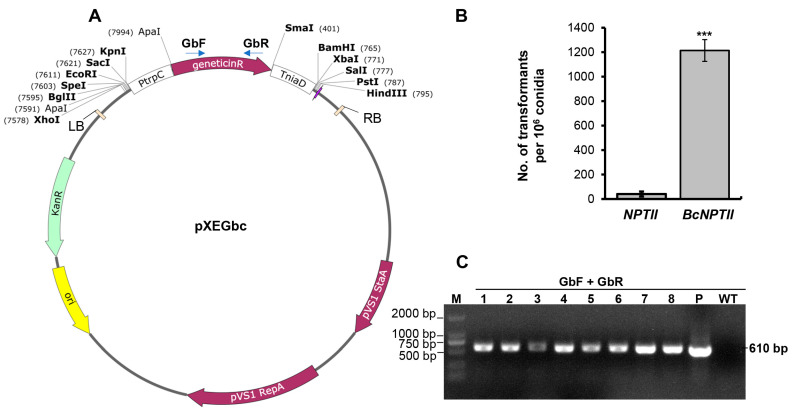
The codon-optimized gene *BcNPTII* significantly enhances the transformation efficiency of *B. cinerea* under the selection pressure of geneticin. (**A**) The binary vector pXEGbc is used for *Agrobacterium tumefaciens*-mediated genetic transformation (ATMT), and it contains *BcNPTII* (geneticinR) controlled by the *PtrpC* promoter and the *TniaD* terminator. (**B**) Quantitative analysis of the transformation efficiency of *BcNPTII* and *NPTII* with the ATMT method. Data represent means ± standard deviations (SD) of three independent experiments. *** indicates statistical significance at *p* < 0.01. (**C**) Identification of randomly selected geneticin-resistant transformants via PCR amplification with specific primers of *BcNPTII*. The templates of lanes 1–8 are transformants (lane 2, transformant B7; lane 4, transformant B9), p and WT indicate the templates are plasmid pXEGbc and the wild-type B05.10, respectively.

To assess the efficiency of the optimized gene *BcNPTII* as a positive selection marker for genetic transformation in *B. cinerea*, we synthesized and cloned this gene into a binary vector pXE [11] under the control of the *trpC* promoter from *Aspergillus nidulans* (*PtrpC*), resulting in the vector pXEGbc (Figure 2A). Similarly, we subcloned the *NPTII* gene used in *M. oryzae* into pXE, also controlled by *PtrpC*, to construct pXEG as a control. Subsequently, we employed the *Agrobacterium tumefaciens*-mediated transformation (ATMT) method to introduce the T-DNA region of both vectors into the *B. cinerea* strain B05.10 and selected transformants using PDA media containing 50 mg/L G418.

The results indicated that *NPTII*, which is suitable for *M. oryzae* transformation, yielded only a small number of transformants for *B. cinerea*, with approximately 41 ± 20 transformants per 10^6^ conidia. However, after codon optimization, the transformation efficiency significantly increased, resulting in approximately 1214 ± 89 transformants per 10^6^ conidia, representing an approximately 30-fold increase in transformation efficiency (Figure 2B). PCR analysis demonstrated that a partial fragment of the codon-optimized gene *BcNPTII* was successfully amplified from eight randomly selected transformants (Figure 2C), confirming that the resistant strains obtained were positive transformants. Additionally, four randomly selected *NPTII* and *BcNPTII* transformants were analyzed for differences in resistance levels to geneticin. It was observed that when the G418 concentration was increased to 200 mg/L, the growth of the *BcNPTII* transformant B7 was significantly faster than that of the *NPTII* transformants, while B9 exhibited slightly faster growth (Figure S2). These results indicate that the codon-optimized gene *BcNPTII* can be used efficiently for random insertion into the genome of *B. cinerea* by generating transformants with varying degrees of increased resistance to geneticin.

### 2.4. The Codon-Optimized Gene BcNPTII Can Be Used for Gene Double Knockout in B. cinerea, Even Using a Field Wild-Type Strain

**Figure 3:**
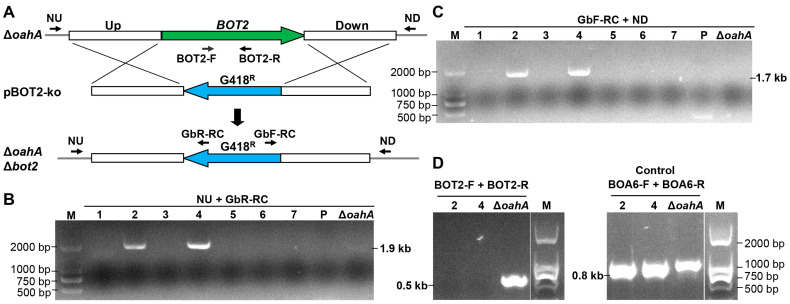
The codon-optimized gene *BcNPTII* was effectively used for *BOT2* knockout from the mutant Δ*oahA* to generate double knockout strains. Δ*oahA* was generated from CLS1, a Chinese field strain. (**A**) The strategy for the generation of the *BOT2* knockout mutant via the gene replacement approach; (**B**–**D**) Identification of the *BOT2* knockout mutant via PCR amplification with the indicated primers by detecting *BOT2* upstream recombination (**B**), detecting downstream recombination (**C**), and detecting *BOT2* loss, with *BOA6* presence as a control (**D**). Numbers 1–7 indicate randomly selected transformants, P indicates plasmid pXEGbc, and Δ*oahA* indicates the deletion mutant of *OAHA* of the CLS1 strain.

One of the most effective methods for deciphering the function of target genes in pathogenic fungi is to knock out the gene and define its function by analyzing the defects of resulting mutants. Given the common occurrence of gene functional redundancy, a reverse genetic strategy through gene replacement to elucidate the function of such genes often requires knocking out two or more functionally redundant genes simultaneously to observe the defective phenotype. This requires the combined use of multiple positive screening markers. To determine whether the optimized *BcNPTII* in this paper can be applied to multiple gene knockouts, we used *BcNPTII* to knock out the second gene *BOT2*, a key gene for the synthesis of the phytotoxin botrydial [19,20], from mutant Δ*oahA*, in which the key gene *OAHA* required for oxalic acid synthesis [21] had been knocked out already by replacement with the hygromycin resistance gene *HPH* (Figure 3A).

Additionally, to confirm whether our optimized geneticin resistance gene can be used for the genetic transformation of field wild-type strains, we performed this double knockout experiment using a field strain, CLS1, isolated from a Chinese greenhouse. Firstly, we replaced the gene *OAHA* in strain CLS1 with *HPH*, resulting in the deletion mutant Δ*oahA*. Subsequently, we introduced the *BOT2* knockout fragment based on *BcNPTII* into this mutant using ATMT. After selection on PDA supplemented with 50 mg/L G418, we randomly picked seven transformants for molecular analysis. Primers NU and GbR-RC were used for detecting upstream homologous recombination at the BOT2 locus in the transformants. The expected recombination would result in a 1.9 kb fragment amplification, while the wild type and knockout vector should not produce any bands. The results showed that transformants #2 and #4 matched the expected pattern (Figure 3B). We also obtained the same results when checking for downstream recombination events using primers GbF-RC and ND; transformants #2 and #4 exhibited the expected gene replacement (Figure 3C), which indicated that *BOT2* was replaced with *BcNPTII* in the two transformants. Furthermore, we amplified an internal fragment of *BOT2* using primers BOT2-F and BOT2-R; the result was consistent with the homologous recombination detection mentioned above. In transformants #2 and #4, *BOT2* was not detected, while the amplification of the control gene *BOA6* was normal (Figure 3D). All of these results indicate that we successfully replaced the *BOT2* gene with the optimized gene *BcNPTII* from the mutant Δ*oahA*, obtaining the double knockout mutant Δ*oahA* Δ*bot2*. Our data suggest that the optimized gene *BcNPTII* can be used in combination with other positive selection markers for gene double or multiple knockouts, even using field wild-type strains.

## 3. Discussion

Many resistance genes or reporter genes that work well in filamentous fungi such as *M. oryzae* often show poor performance when directly used in *B. cinerea*, mainly due to the substantial differences in codon usage preference between *B. cinerea* and other filamentous fungi such as *M. oryzae* [18]. In this study, according to the codon usage preference of *B. cinerea*, we optimized the codons of the geneticin resistance gene and found it can be effectively used as a positive selection marker for *B. cinerea* genetic transformation.

We found that *B. cinerea* was more sensitive to G418, and 50 mg/L G418 completely inhibited the conidial germination or the mycelium growth of *B. cinerea* compared to the higher G418 concentration used in positive selection against other fungi, e.g., 400 mg/L for *M. oryzae* [15], 250 mg/L for *Paecilomyces lilacinus* [22], and 200 mg/L for *Ashbya gossypii* [23] and *Fusarium oxysporum* [24]. We recommend 50–75 mg/L G418 for the genetic transformation of *B. cinerea*.

Our study showed that using the optimized *BcNPTII* as a positive selection marker for transforming *B. cinerea* resulted in a much higher number of transformants, with an approximately 30-fold increase in transformation efficiency. Due to the substantial differences in codon usage preference between *M. oryzae* and *B. cinerea* (Table S1), the expression level of *NPTII* used for the rice blast fungus was too low to withstand selection pressure when used to transform *B. cinerea*, resulting in very few transformants growing on the selection plates. Only when the expression of *NPTII* reached a certain level could the transformants grow under selection pressure. In addition to codon preference, the expression level of exogenous genes in transformants is also influenced by the insertion site in the genome. When the gene is inserted into regions containing enhancers nearby, the expression level of the positive selection marker can reach a level that allows the transformants to overcome selection pressure and be selected. This explains the phenomenon that, even when using the not codon-optimized gene *NPTII*, a small number of transformants could still be obtained despite the low transformation efficiency. Nevertheless, when the selection pressure increased, the resistance level of the transformants containing the optimized gene generally showed varying degrees of improvement.

To simultaneously knock out multiple genes in a strain, sexual mating can be used to gather single-gene deletions into the same descendant strain [1]. However, due to the complex nature of sexual reproduction and tetrad analysis in filamentous fungi, especially in heterothallic fungi like *B. cinerea*, this method is relatively inefficient. Recently, with the rapid development of gene editing technology, a single positive selection marker loaded onto a mini-chromosome in *B. cinerea* allows for the sequential knockout of target genes, resulting in strains with multiple gene knockouts [14]. However, considering the multinucleate nature of *B. cinerea*, this strategy sometimes has low efficiency in obtaining homozygous mutants [25], in addition to carrying a certain degree of off-target risk, and requires further optimization and improvement. Presently, for simultaneously knocking out multiple genes in *B. cinerea*, the classical method using different positive selection markers to individually knock out target genes through homologous recombination remains one of the most efficient ways. This study contributes new and useful positive selection markers for multi-gene knockout in *B. cinerea*.

The codon-optimized geneticin resistance gene *BcNPTII* reported in this paper can be applied to the construction of random insertional mutant libraries of *B. cinerea*, the expression of exogenous functional genes, reporting genes such as GFP, and genetic complementation. It can also be used for gene knockout, especially in combination with other selection markers for double or multiple gene knockouts. Both model strains and field isolates of *B. cinerea* were efficiently transformed. Notably, we used *PtrpC* instead of *POlic* to drive *BcNPTII* to reserve the strong promoter *POlic* for the overexpression of reporter genes such as GFP, ensuring sufficient expression levels to meet the needs of related research. In summary, the codon-optimized *BcNPTII* can be efficiently applied to the analysis of the pathogenesis of *B. cinerea*, which will help to identify more alternative molecular targets for disease control. Since the codon usage preferences of *Sclerotinia sclerotiorum* and *B. cinerea* are very similar [10], it is speculated that the optimized *BcNPTII* described in this paper can be used as a positive selection marker gene for genetic transformation in *S. sclerotiorum*. With the use of the similar codon-optimization method, other low-efficiency positive selection markers, such as *BAR*, *IVL1*, etc., may also be improved for the efficient genetic transformation of the grey mould fungus.

## 4. Materials and Methods

### 4.1. Botrytis cinerea Strains and Culture Conditions

The *B. cinerea* strains used in this study are listed in Table 1. Wild-type strain B05.10 and a Chinese field strain CLS1 were used in genetic modifications or as control strains. All strains were cultivated and maintained on potato dextrose agar (PDA) plates as previously described [11].

### 4.2. Synthesis of the Codon-Optimized Geneticin Resistance Gene and Construction of the Random Insertion Binary Vector

The codon-optimized version of *B. cinerea NPTII* (*BcNPTII*) was synthesized by GENEWIZ (Suzhou, China) and was cloned into the pUC57 cloning vector via *Eco*R V. *Kpn* I and *Bam*H I sites were included during synthesis for the generation of the expression constructs (see below). For transformation, the coding sequence of *BcNPTII* was digested with *Kpn* I and *Bam*H I and subcloned into pXE, a binary vector used for fungal genetic transformation [11], to generate pXEGbc (Table 2). For comparative analysis, the *NPTII* expression cassette used for *Magnaporthe oryzae* transformation [17] was subcloned into pXE by digestion with *Kpn* I and *Sal* I and subsequent ligation to generate pXEG (Table 2).

### 4.3. Construction of the Knockout Vector

The method for constructing a knockout vector for gene *BOT2* of *B. cinerea* is as follows. With the use of primers BOT2-UP-F/BOT2-UP-R and B05.10 genomic DNA as a template, a 1020 bp fragment upstream of *BOT2* was amplified with Q5 high-fidelity DNA polymerase (NEB, Ipswich, MA, USA). With the use of primers BOT2-DN-F/BOT2-DN-R (Table 3), a 959 bp fragment downstream of *BOT2* was amplified. The NEBuilder HiFi DNA Assembly Cloning Kit (NEB, Ipswich, MA, USA) was then used to clone the above two DNA amplification products via recombination into the respective *Hin*d III and *Kpn* I sites of the pXEGbc vector, with the codon-optimized *BcNPTII* in between, to construct the knockout vector pBOT2-ko, which was subsequently confirmed by sequencing.

### 4.4. Genetic Transformation of B. cinerea

The random insertion binary vectors pXEG and pXEGbc, or the knockout vector pBOT2-ko, were transformed into the *Agrobacterium tumefaciens* strain AGL-1, and *Agrobacterium tumefaciens*-mediated transformation (ATMT) was used to obtain fungal transformants following the methods reported previously [27,28,29]. Transformants were selected using PDA media containing 50 mg/L G418.

### 4.5. Molecular Analysis of Transformants

Isolation of *B. cinerea* genomic DNA was performed according to a documented protocol [30]. The *BOT2* knockout strains were screened by PCR amplifications as follows. Primers NU/GbR-RC and GbF-RC/ND (Table 3) were used to detect whether homologous recombination had occurred upstream and downstream, respectively, of the target gene. Primers BOT2-F/BOT2-R (Table 3) were used to determine if the target gene was no longer present in the genome.

### 4.6. Sensitivity Analysis of B. cinerea to Geneticin

To test the sensitivity of *B. cinerea* to geneticin, G418 concentration gradient experiments were performed. Briefly, mycelial plugs of B05.10 were inoculated on PDA plates supplemented with G418 at concentrations of 0, 1, 10, and 25 mg/L. To test the conidial sensitivity to geneticin, two μL 2 × 10^5^ mL^−1^ conidial suspensions of B05.10 were inoculated on PDA plates supplemented with G418 at concentrations of 0, 5, 10, 15, 20, and 50 mg/L. Colony growth was assayed at 3–4 days post inoculation (DPI). To test the ability of geneticin to suppress conidial germination, ten μL 2 × 10^5^ mL^−1^ conidial suspension in PDB media supplemented with or without 50 mg/L G418 was inoculated on slides and observed at 12 h post inoculation (HPI).

The size (diameter or radius) of the colonies grown under G418 pressure was used to reflect the sensitivity of the strain to G418. Under the same G418 concentration, the smaller the colony size, the more sensitive the strain to G418. The resistance level of a strain was reflected by calculating the percentage colony size with G418 compared to that without G418. Colonies grown on PDA plates supplemented with different concentrations of G418 were photographed, and their diameter or radius was measured and calculated using ImageJ software v1.52v (https://imagej.en.softonic.com/download (accessed on 15 May 2023)).

## Figures and Tables

**Table 1:** Strains of *Botrytis cinerea* used in this paper.

Strain	Description	Source
B05.10	Wild-type strain	[26]
CLS1	A Chinese field strain (Wild-type strain)	This study
N2	*NPTII* random insertion in B05.10	This study
N4	*NPTII* random insertion in B05.10	This study
B7	*BcNPTII* random insertion in B05.10	This study
B9	*BcNPTII* random insertion in B05.10	This study
Δ*oahA*	*BcOAHA* gene knockout (Δ*BcoahA::HPH*) in CLS1	This study
Δ*oahA* Δ*bot2*	Double gene knockout (Δ*BcoahA::HPH*; Δ*Bcbot2::BcNPTII*) in CLS1	This study

**Table 2:** Vectors used in this paper.

Vector	Description	Source
pXEH	Binary vector used for knockout of fungal genes, containing the *HPH* gene within its T-DNA region; Km^R^	[11]
pXEG	Binary vector used for knockout of fungal genes, containing the *NPTII* gene within its T-DNA region; Km^R^	This study
pXEGbc	Binary vector used for knockout of fungal genes, containing the *BcNPTII* gene within its T-DNA region; Km^R^	This study
pBOT2-ko	Binary vector used for knockout of *BcBOT2*, containing the *BcNPTII* gene within its T-DNA region; Km^R^	This study

**Table 3:** Primers used in this paper.

Name	Sequence (5′–3′)
**For identification of *BcNPTII***	
GbF	GGCACAACAGACAATCGG
GbR	ATCACGGGTAGCCAAAGC
**For construction of the *BOT2* knockout vector**	
BOT2-UP-F	tgggaattcgagctcggtacAGGTCCTGTTGACATGGAATC
BOT2-UP-R	agtcgacctgcaggcatgcaCGGAACACGAACGAATGG
BOT2-DN-F	ccttcaatatcagttggtacTGGGCTAAGCACAGGACAATG
BOT2-DN-R	aatgcggctccacagctgcaCCTCCCGATGATAAGGGTTC
**For Screening the *BOT2* knockout strains**	
NU	TGGACAACCTTGAGGACGAG
ND	GCGGAGTTGCCTTGGAGT
GbF-RC	CCGATTGTCTGTTGTGCC
GbR-RC	GCTTTGGCTACCCGTGAT
**For identification of *BOT2***	
BOT2-F	GACTGGAATCATTGGGTTTG
BOT2-R	GCGTTTGTGCTGGTCTATC
**For identification of *BOA6* (to confirm the genomic DNAs of transformants were suitable for amplification)**	
BOA6-F	AACTCGGCAATCGAACCT
BOA6-R	GCAGCATAAGCAGCACCA

## Data Availability

The data presented in this study are available on request from the corresponding author.

## Supplementary Materials

The following supporting information can be downloaded at https://www.mdpi.com/article/10.3390/plants13020324/s1, Figure S1: Quantification of the radial growth (in diameter) of B05.10 at 3 days post inoculation (DPI) on PDA plates supplemented with different concentrations of G418; Figure S2: The transformants derived from the codon-optimized gene *BcNPTII* show varying degrees of increased resistance to geneticin; Table S1: Codon usage of fungal genes and the two geneticin resistance genes.
