# Supplementary material for: Codon Optimization Enables the Geneticin Resistance Gene to Be Applied Efficiently to the Genetic Manipulation of the Plant Pathogenic Fungus Botrytis cinerea
Source: Plants (Basel). 2024 Jan 22;13(2):324. doi: 10.3390/plants13020324 (PMC10821057; doi:10.3390/plants13020324)
Supplement: Supplementary file 1 [file plants-13-00324-s001.zip › plants-2811390-supplementary.pdf]

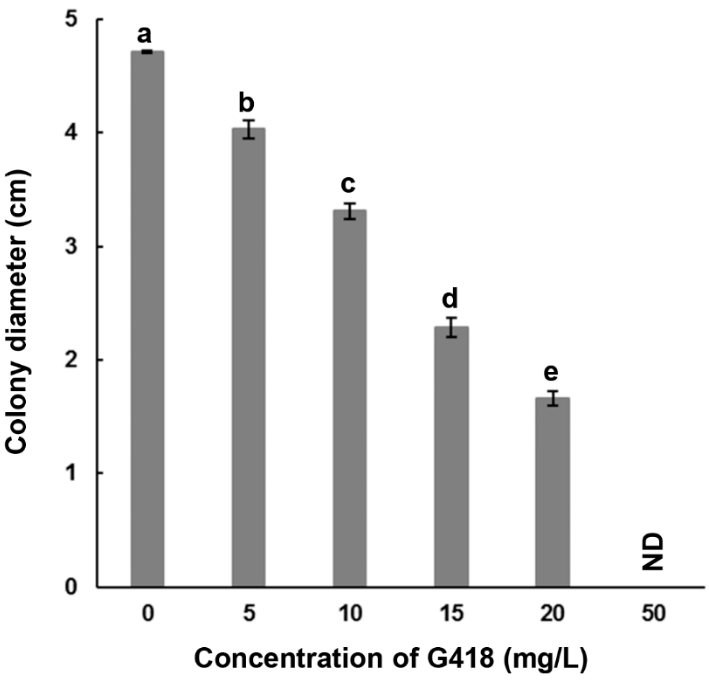

Figure S1. Quantification of the radial growth (in diameter) of B05.10 at 3 days post inoculation (DPI) on PDA plates supplemented with different concentrations of G418. ND: Not detected. Data represent means ± standard deviations (SD) from three independent experiments in which triplicate colonies were analyzed for each treatment. The letters above columns indicate significant differences analyzed with SPSS software at $p<0.05$.

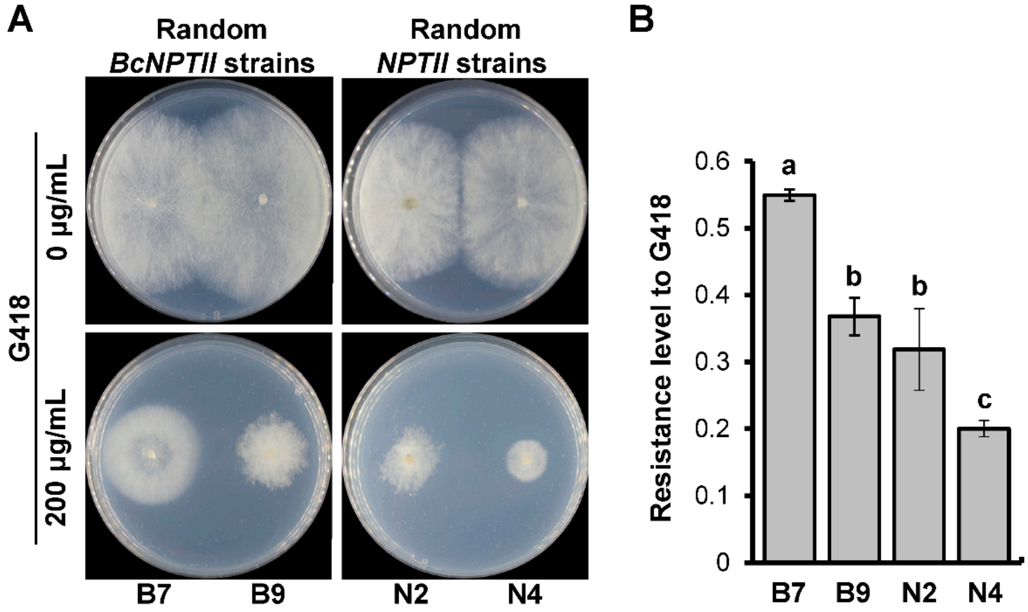

Figure S2. The transformants derived from the codon-optimized gene *BcNPTII* show varying degrees of increased resistance to geneticin. (**A**) Mycelial plugs of 4 randomly selected geneticin-resistant transformants were inoculated on PDA plates supplemented with or without G418. Photographs were taken at 2 DPI. (**B**) Quantification of the resistance levels of 4 randomly selected transformants derived from different resistance genes. The resistance level of a strain is reflected by calculating the percentage of the colony size with G418 compared to that without G418. The letters above columns indicate significant differences analyzed with SPSS software at $p<0.05$. B7 and B9 were derived from *BcNPTII* transformation; N2 and N4 were derived from *NPTII* transformation.

Table S1. Codon usage of fungal genes and the two geneticin resistance genes

Parameter		Value for indicated organism or gene[a]							
		M. oryzae	*N. crassa*	*A. gossypii*	*F. oxysporum*	*S. sclerotiorum*	*B. cinerea*	*NPTII*	*BcNPTII*
Coding GC content (%)		56.3	56.1	52.8	51.2	47.6	46.6	59%	48%
Codon usage (%)									
Ala									
	GCT	19.9	24.3	19.0	39.5	38.2	34.2	26(9)	40(14)
	GCC	42.8	41.4	25.4	40.2	26	24.1	34(12)	29(10)
	GCA	18.1	14.5	21.8	7.9	27.4	29.6	17(6)	31(11)
	GCG	19.3	19.9	33.8	12.4	8.3	12	**23(8)**	0
Arg									
	AGA	9.3	12.8	15.7	20.2	27.9	27.1	0	40(8)
	AGG	18.5	19.1	14.2	6.0	10.8	10.2	15(3)	0
	CGT	14.6	14.4	12.5	15.5	23.2	20.2	10(2)	25(5)
	CGC	30.9	28.5	28.4	35.2	13.8	14.6	**40(8)**	0
	CGA	12.8	11.4	6.5	23.2	19.8	21.1	5(1)	35(7)
	CGG	13.9	13.8	22.7	0.0	4.4	6.8	**30(6)**	0
Asn									
	AAT	23.8	27.7	39.1	17.2	43.4	51.9	100(3)	67(2)
	AAC	76.2	72.4	60.9	82.8	56.6	48.1	0	33(1)
Asp									
	GAT	35.6	42.4	42.1	44.1	69.2	66.5	48(12)	68(17)
	GAC	64.4	57.6	57.9	55.9	30.8	33.5	52(13)	32(8)
Cys									
	TGT	27	30.3	35.8	31.7	46.7	53.1	20(1)	60(3)
	TGC	73	69.7	64.2	68.3	53.3	46.9	80(4)	40(2)
Glu									
	GAA	29.8	34.5	36.9	17.0	53.1	54.7	56(10)	56(10)
	GAG	70.2	65.5	63.1	83.0	46.9	45.3	44(8)	44(8)
Gln									
	CAA	36	39.4	29.1	19.2	71.8	67.7	27(3)	73(8)
	CAG	64	60.6	70.9	80.8	28.2	32.3	**73(8)**	27(3)
Gly									
	GGT	23.4	25.5	21.9	35.4	43.7	35.7	24(5)	43(9)
	GGC	45.8	40.4	40.1	44.8	16.1	20.9	**48(10)**	14(3)
	GGA	18	18.9	15.0	17.5	34.3	33.4	14(3)	43(9)
	GGG	12.7	15.2	23.1	2.3	6	10.1	14(3)	0
His									
	CAT	31.8	39.0	41.7	48.4	50.2	58.2	57(4)	57(4)
	CAC	68.2	61.0	58.3	51.6	49.8	41.8	43(3)	43(3)

Continued on the following page

Parameter		Value for indicated organism or gene[a]							
		M. oryzae	*N. crassa*	*A. gossypii*	*F. oxysporum*	*S. sclerotiorum*	*B. cinerea*	*NPTII*	*BcNPTII*
Ile									
	ATT	30.5	31.4	33.9	37.6	38.3	44.8	20(2)	60(6)
	ATC	56.4	59.4	43.1	61.4	51.3	39.6	70(7)	40(4)
	ATA	13	9.2	23.0	1.1	10.4	15.6	10(1)	0
Leu									
	TTA	3.7	3.3	8.2	3.1	11.4	11.4	0	0
	TTG	15.3	18	19.4	20.3	23.5	23.2	13(4)	28(9)
	CTT	15.2	17.2	12.6	22.3	25.4	24.4	25(8)	59(19)
	CTC	30.3	32.3	15.8	34.9	25.5	20.7	19(6)	13(4)
	CTA	6.4	7.2	13	2.3	6.5	9.5	9(3)	0
	CTG	28.9	22	31	17.2	7.7	10.7	**34(11)**	0
Lys									
	AAA	24.7	22.5	31.0	7.5	37.3	45.7	50(2)	50(2)
	AAG	75.3	77.6	69.0	92.5	62.7	54.3	50(2)	50(2)
Phe									
	TTT	38.4	34.8	42.2	21.9	30.8	41.8	18(2)	55(6)
	TTC	61.6	65.2	57.8	78.1	69.2	58.2	82(9)	45(5)
Pro									
	CCT	21	23.4	20.7	36.9	34.6	31.6	18(2)	45(5)
	CCC	31.8	34.8	21.9	39.0	14	17.9	18(2)	0
	CCA	21.1	19.2	25.8	19.4	42.3	39.1	18(2)	55(6)
	CCG	26.1	22.6	31.6	4.7	9.1	11.4	**45(5)**	0
Ser									
	AGT	8.3	10.6	10.1	10.3	13.7	16.3	0	20(2)
	AGC	24.9	21.3	20.8	23.2	12.8	12.9	0	0
	TCT	13.6	14.6	17.2	23.7	22.5	22.8	40(4)	40(4)
	TCC	18	24.4	18.4	25.1	26.4	16.6	20(2)	20(2)
	TCA	12.5	11.3	11.9	7.6	14.7	19.4	20(2)	20(2)
	TCG	22.7	17.7	21.6	10.0	9.9	12	20(2)	0
Thr									
	ACT	17.4	18.6	20.6	39.6	33.9	31.6	20(2)	40(4)
	ACC	38	41.1	26.8	40.5	37.5	27.1	40(4)	20(2)
	ACA	19.6	17.9	22.2	12.7	19.6	28.8	10(1)	40(4)
	ACG	25	22.5	30.3	7.2	9	12.4	**30(3)**	0
Tyr									
	TAT	27.6	32.7	35.6	38.2	45.7	49.3	75(3)	50(2)
	TAC	72.4	67.3	64.4	61.8	54.3	50.7	25(1)	50(2)
Val									
	GTT	21.8	23.2	21.3	37.6	38.4	36.7	19(3)	50(8)
	GTC	43.5	41.7	24.4	48.5	38.3	31.5	25(4)	44(7)
	GTA	8.9	9.1	13.4	1.1	12	16.2	6(1)	6(1)
	GTG	25.8	26.0	40.9	12.8	11.2	15.6	**50(8)**	0

[a] Numbers in parentheses indicate codon numbers in geneticin resistance genes. Bold numbers indicate codons mainly used in *NPTII* but underrepresented in B. cinerea and S. sclerotiorum

compared to other fungi. GC content and codon frequencies of fungal coding sequences were taken from http://www.kazusa.or.jp/codon/. The fungi analyzed were as follows: *Magnaporthe oryzae* (*M. oryzae*), *Neurospora crassa* (*N. crassa*), *Ashbya gossypii* (*A. gossypii*), *Fusarium oxysporum* (*F. oxysporum*), *Sclerotinia sclerotiorum* (*S. sclerotiorum*), *Botrytis cinerea* (*B. cinerea*).

Note： A site for the commonly used restriction enzyme *Pst* I was eliminated from the final sequence of *BcNPTII*.
